# Commentary: Corroboration of a Major Role for Herpes Simplex Virus Type 1 in Alzheimer's Disease

**DOI:** 10.3389/fnagi.2018.00433

**Published:** 2019-01-10

**Authors:** Janusz K. Rybakowski

**Affiliations:** Department of Adult Psychiatry, Poznan University of Medical Sciences, Poznan, Poland

**Keywords:** Alzheimer's disease, HSV1, herpes simplex virus type 1, lithium, labial herpes, cognition

In her review paper, Dr. Ruth Itzhaki provides evidence that infection with herpes simplex virus type 1 (HSV1) makes a significant risk factor for Alzheimer's Disease (AD). She speculates that the use of antiviral drugs may decrease such a risk. In connection with this, it could be reminded that lithium is a drug, which anti-herpes properties have been documented both in experimental and clinical studies (Skinner et al., 1980; Rybakowski and Amsterdam, 1991) and there is both experimental, epidemiological, and clinical evidence showing that lithium can exert a prophylactic and therapeutic effect on the AD (Rybakowski, 2018). Therefore, a hypothesis can be put forward that anti-herpes activity of lithium can make a link to a possible prophylactic and therapeutic action of this ion in the AD.

Nearly four decades ago researchers from the University of Birmingham showed that lithium in 5–30 mmol/l concentration inhibits replication of the herpes simplex virus. They suggested that mechanism of action may involve a blockage of synthesis of the virus's DNA by lithium or competition with magnesium ions catalyzing enzymatic reactions of the virus (Skinner et al., 1980). At that time also descriptions of cases of labial herpes remissions while using lithium appeared (Gillis, 1983). Labial herpes is due to an infection of herpes simplex virus type 1 (HSV-1), occurs in ~1/3 of the population, and its course is characterized by frequent recurrences. Retrospective research of labial herpes recurrences in patients receiving lithium for prophylactic purposes was carried out in a collaborative Polish-American study. There were 69 patients in the Polish group, treated with lithium for a mean of 8 years, including 28 persons with recurrent labial herpes. Among the latter, during lithium prophylaxis, in 13 patients (46%) there was full cessation of recurrence of herpes, among seven the frequency of recurrences decreased, among six it remained at the same level and among two it increased. The general decrease of recurrence frequency was 64%. Better prophylactic result occurred in patients in whom the lithium concentration in the serum was higher than 0.65 mmol/l, and in the red blood cells exceeded 0.35 mmol/l. In the American group, there were two subgroups, age- and sex-matched. In each group there were 21 men and 31 women, aged on average 45 years. The mean duration of pharmacological treatment was 5 years in both groups. In the first bipolar group, lithium was used, whereas the second group of depressed patients was treated with antidepressant drugs. Compared with the preceding 5-year period, labial herpes recurrences were reduced in the lithium group by 73%. However, no significant difference occurred in the antidepressant-treated patients (Rybakowski and Amsterdam, 1991). In another study, Amsterdam et al. (1990) in a placebo-controlled trial showed that chronic lithium administration might prevent recurrent genital herpes infections.

The evidence has been acquired in recent decades for a possible effect of lithium on pathogenic changes of the AD. Such an effect has been mostly assigned to the inhibition by lithium of glycogen synthase kinase-3 (GSK-3). This enzyme is important in the metabolism of amyloid precursor protein and the phosphorylation of the tau protein, the leading factors in the pathogenesis of the AD. Experimental studies on rats, using cortical and hippocampal neurons, showed that lithium treatment decreases the GSK3 mRNA (Mendes et al., 2009). In mutant tau transgenic mice having severe neurofibrillary disruption, lithium can delay the progress of neurofibrillary tangles (Leroy et al., 2010). In the AD model of *Drosophila* fly, the GSK-3 inhibition by lithium alleviates the pathology of the amyloid-beta (Sofola et al., 2010).

The association between lithium treatment and a decreased risk of dementia was found in big cohorts and many case-control studies (Donix and Bauer, 2016). Kessing et al. (2008) using the Danish nationwide register of lithium prescriptions showed that in persons with chronic lithium use, the level of dementia was similar as in the general population. On the other hand, in persons receiving anticonvulsant drugs, the dementia risk raised with the length of treatment. These researchers also demonstrated that in patients with bipolar disorder, long-term lithium treatment decreased the rate of dementia, what was not the case for long-term administration of anticonvulsant, antidepressant, or antipsychotics drugs (Kessing et al., 2010). Encouraging outcomes have been obtained in some studies where lithium was employed as a therapy for dementia. Matsunaga et al. (2015) made a meta-analysis of clinical trials including 232 participants and concluded that lithium was significantly better than placebo in reducing a cognitive impairment.

In patients with bipolar disorder, infection with herpes simplex virus type 1 was associated with impaired cognitive functioning (Dickerson et al., 2004). In my review on the effect of lithium on neurocognitive functioning (Rybakowski, 2016) I suggested that specific effect of lithium against HSV-1 infection in patients with bipolar disorder may be a contributing factor to its favorable effect on cognition in this disorder. In this commentary, a similar hypothesis can be put forward for the AD, linking the anti-herpes activity of lithium with its possible prophylactic and therapeutic action in the AD.

To conclude, let me mention two interesting recent papers where a connection between lithium level in drinking water and dementia was investigated. Kessing et al. (2017) performed nationwide, case-control research in Denmark. They showed that the frequency of dementia was lower in subjects exposed to more than 15.0 and 10.1–15.0 μg/L of lithium in drinking water, and higher in those with the levels of 5.1–10.0 μg/L. Comparable patterns in this respect were observed for both Alzheimer disease and vascular dementia. In Texas, Fajardo et al. (2018) examined a connection between the concentration of lithium in drinking water and AD mortality. They found that differences in AD mortality showed a negative correlation with drinking water lithium concentrations. Their results also suggest that such important risk factors for the AD as obesity and diabetes, type 2, were inversely associated with lithium levels in drinking water.

## Author Contributions

The author confirms being the sole contributor of this work and has approved it for publication.

### Conflict of Interest Statement

The author declares that the research was conducted in the absence of any commercial or financial relationships that could be construed as a potential conflict of interest.
